# CCT7 Expression Affects the Prognosis of Lung Adenocarcinoma

**DOI:** 10.1002/cnr2.70610

**Published:** 2026-06-29

**Authors:** Yue Li, Longqian Wei, Guiyu Feng, Guosheng Li, Jielin Ma, Xiang Gao, Jun Liu, Nuo Yang, Huafu Zhou

**Affiliations:** ^1^ Department of Cardiothoracic Surgery The First Affiliated Hospital of Guangxi Medical University Nanning Guangxi China; ^2^ Department of Anesthesiology The People's Hospital of Guangxi Zhuang Autonomous Region Nanning Guangxi China

**Keywords:** CCT7, lung adenocarcinoma, prognostic value

## Abstract

**Background:**

CCT7 is a chaperonin containing tailless complex polypeptide 1 subunit. The study focused on investigating its prognostic significance for lung adenocarcinoma (LUAD).

**Methods:**

CCT7 expression in LUAD and matched non‐carcinoma lung samples was examined via TCGA database. Besides, we utilized the GEO database and RT‐qPCR for validating differential CCT7 expression. The relation with clinical factors and its functions in diagnosis and prognosis prediction were determined. GO, KEGG, along with GSEA were conducted for exploring CCT7's functions. We also performed CIBERSORT, ssGSEA, and ESTIMATE for examining the LUAD immune microenvironment. By analyzing tumor mutational burden (TMB) and immune checkpoints, the relationship between CCT7 and immunotherapy was explored.

**Results:**

CCT7 expression significantly increased within LUAD relative to matched non‐carcinoma lung samples. As revealed by GO, KEGG, as well as GSEA results, CCT7 was mostly related to cell movement and regulation of multiple biological processes. Furthermore, CCT7 expression varied significantly among different immune cells, and those having increased CCT7 expression showed greater TMB.

**Conclusions:**

An increase in CCT7 expression carries significant implications for diagnosing and predicting LUAD prognosis.

## 1 Background

Lung cancer is the neoplasm with highest prevalence worldwide, characterized by great incidence and mortality rates. It is also the major factor causing cancer‐associated mortality in female populations across regions such as North America, Europe, and Australia [1]. It is categorized as lung squamous cell carcinoma (LUSC), lung adenocarcinoma (LUAD), or small cell lung cancer (SCLC), with LUSC and LUAD collectively referred to as non‐small cell lung cancer (NSCLC), occupying about 85% of lung cancers [2]. Most lung cancer patients show non‐specific early symptoms; as a result, the diagnosis is often made at an advanced stage. For stage IA patients, their 5‐year survival rate reaches up to 90%, but it is below 10% in those with stage IV disease, making early and effective screening and diagnosis important for predicting patient prognosis [3].

The chaperonin containing tailless complex polypeptide 1 (CCT), referred to as the tailless complex polypeptide 1 ring complex (TRiC), represents the important eukaryotic molecular chaperone with eight subunits, involved in cytoskeleton generation and tubulin folding [4]. Tumor cell proliferation, invasion, and migration are primarily driven by movement of tubulin and cytoskeleton proteins actin; thus, CCTs fundamentally influence cancer genesis and progression [5]. As such, CCTs may become potential targets for inhibiting tumor proliferation and invasion. CCT7, one of the subunits of CCT, participates in processes such as autophagy and the movement of fibroblasts and serves as a potential marker unique to fibroblast motility [6]. Studies have suggested that CCTs are overexpressed within head and neck squamous cancer (HNSC) and are related to unfavorable prognostic outcomes of patients; CCT7 expression is even higher among advanced HNSC patients, demonstrating that CCT7 may be the candidate marker for diagnosing HNSC [7]. However, its effects on other tumors, especially in lung cancer, remain unclear. Against this background, to explore the role of CCT7 in LUAD, the study assessed the diagnostic and prognostic value of CCT7 in patients with LUAD. Additionally, the current study analyzed the function of CCT7 and investigated its role in the immunology of LUAD.

## 2 Methods

### 2.1 CCT7 Expression Analysis in TCGA, GEO, and HPA Databases

We obtained data for 601 LUAD samples based on The Cancer Genome Atlas (TCGA), encompassing 541 LUAD and 59 matched non‐carcinoma samples, along with relevant clinical data (age, sex, pathological stage, TNM classification) and survival data (survival status, overall survival time), total 508 LUAD cases having sufficient survival data. Data analysis was conducted using FPKM data for gene expression. Patients with LUAD were categorized as high or low CCT7 expression group based on median CCT7 level. CCT7 expression was validated by using of Gene Expression Omnibus database (GEO, https://www.ncbi.nlm.nih.gov/geo/). GSE31210 dataset includes gene expression data for 226 LUAD along with 20 matched non‐carcinoma lung samples, all participants aged between 30 to 89 years old and had histologically confirmed primary LUAD, with detailed medical history information provided. We utilized Human Protein Atlas (HPA, https://www.proteinatlas.org/) database for obtaining immunohistochemical staining results for CCT7 protein within non‐carcinoma lung tissues and LUAD samples.

### 2.2 Relation of CCT7 Expression With Survival of LUAD Patients

LUAD samples from TCGA dataset were grouped according to different clinical characteristics, and compared CCT7 expression differences between groups. The capability of CCT7 as the biomarker for diagnosing and predicting LUAD prognosis was evaluated by constructing Receiver Operating Characteristic (ROC) as well as Kaplan–Meier (KM) curves. Both R “survival” (version 35‐7) and “Survminer” (version 0.4.9) packages were employed for conducting KM analysis. Cox regression or log‐rank tests were used for analyzing the relation of CCT7 expression with survival rate in LUAD patients and evaluated how different CCT7 expression affected prognosis within different clinical characteristics.

### 2.3 Screening and Functional Analysis of CCT7 Differentially Expressed Genes Within LUAD

An analysis on TCGA‐LUAD samples was performed for identifying differentially expressed genes (DEGs) associated with CCT7 expression. Volcano plots were utilized to visualize DEGs. The identified 5194 DEGs (|logFC| ≥ 1.0, *p*_adj_ < 0.05) (including 4095 upregulated DEGs and 1099 downregulated DEGs) were conducted Gene Ontology (GO), Kyoto Encyclopedia of Genes and Genomes (KEGG), along with Gene Set Enrichment Analysis (GSEA) functional analysis with R clusterProfiler package (version 4.10.0). GO and KEGG results were presented as bar graphs, while GSEA displayed those top 10 functions associated with DEGs.

### 2.4 Constructing and Evaluating a Predictive Nomogram

By using the R “rms” package (version 6.7‐1), a nomogram that included age, sex, TNM stage, and CCT7 expression was established for predicting overall survival (OS) in LUAD patients. Nomogram homogeneity in forecasting different OS outcomes was assessed based on calibration curves. Harrell's concordance index was utilized to generate the c‐index. We utilized the area under the curve (AUC) for assessing the nomogram's predictive performance.

### 2.5 Gene Correlation Analysis and Hub Gene Prediction

Using Pearson's test, we analyzed genes related to CCT7 within the TCGA‐LUAD cohort, selecting the 20 most associated genes (10 with positive whereas 10 with negative correlations) based on a correlation coefficient |*r*| > 0.4 and *p* < 0.05 as the selection threshold. STRING (https://string‐db.org/) is a database designed to integrate all known and predicted protein–protein interactions (PPI) [8]. In this study, it was used to analyze the interactions among proteins encoded by CCT7 and related genes. Survival analysis of the selected related genes from the PPI network was performed by KM analysis to investigate the relation of CCT7‐associated genes with prognostic outcomes in LUAD cases.

### 2.6 CCT7 Expression and Tumor Immune Infiltration

ESTIMATE can infer the immune/stromal cell proportion within tumor samples according to gene expression characteristics, which can further assess tumor purity [9]. ESTIMATE was used to assess the tumor microenvironment composition of all LUAD samples. By adopting the R “estimate” package (version 1.0.13), stromal/immune/ESTIMATE scores and tumor purity for all LUAD samples were determined. CIBERSORT, the deconvolution algorithm, performs immune cell infiltration analysis through deconvoluting gene expression microarray data, was used for determining proportions of 22 immune cell subtypes within tumor tissues [10], with all proportion scores for one sample were 1. We utilized R “CIBERSORT” package (version 0.1.0) for comparing different immune cell proportions in high versus low CCT7 expression groups. This study conducted ssGSEA analysis (with “GSVA” package, version 1.50.0) to explore infiltration of 28 immune cell types within LUAD samples between these two groups.

### 2.7 Analysis of CCT7 Expression With Tumor Mutation Burden and Immune Checkpoint Expression

Simple nucleotide mutation data of 526 LUAD cases were collected based on TCGA database; meanwhile, waterfall plots of tumor mutations for both high CCT7 expression group and low CCT7 expression group were drawn with R “maftools” package (version 2.18.0). These plots show the 30 genes exhibiting greatest mutation proportions within LUAD samples. Subsequently, differences in the levels of TMB and 11 immune checkpoint levels were compared between these two groups. These immune checkpoints have been mentioned in previous research [11].

### 2.8 Patient and Sample Collection

Fresh tumors and matched non‐carcinoma samples were harvested in 20 LUAD patients receiving surgical lung resection from September to November 2023 from the Department of Cardiothoracic Surgery of the First Affiliated Hospital of Guangxi Medical University. Patients did not receive chemotherapy, radiotherapy, or additional adjuvant therapy preoperatively. Tumor samples were obtained at the non‐necrotic center of the surgically removed tumors, and the matched non‐carcinoma samples from at least 5 cm from the tumor edge. After removal, the samples were put into enzyme‐free cryotubes at once and preserved in a −80°C freezer within half an hour. Our protocol was granted approval by the Ethics Committee of The First Affiliated Hospital of Guangxi Medical University (no. 2024‐E338‐01). The subjects and their legal guardians provided informed consent.

### 2.9 RNA Isolation and Reverse Transcription

The RNA extraction kit (Monad, China) was adopted for isolating total tissue RNA in line with specific protocols. Then, RNA (1.0 μg) was prepared into cDNA through reverse‐transcription with Prime Script RT Master Mix (Takara, Japan).

### 2.10 Real‐Time Quantitative PCR

2 × RealStar Fast SYBR qPCR Mix (Genstar, China) was utilized in real‐time quantitative PCR (RT‐qPCR) using the Applied Biosystems 7500 Real Time PCR system (Thermo, USA) for measuring gene levels. CCT7 and control gene levels were examined using the 2^−ΔΔCt^ approach. Primer sequences utilized included:

CCT7‐F: 5′‐GCAGTTTATGGAGGAGACA‐3′.

CCT7‐R: 5′‐CCTGGAATAGTCCTTGAGT‐3′.

GAPDH‐F: 5′‐CAGGAGGCATTGCTGATGAT‐3′.

GAPDH‐R: 5′‐GAAGGCTGGGGCTCATTT‐3′ (Genecreate, China).

### 2.11 Multiplex Immunofluorescence Staining

Multiplex immunofluorescence (mIF) staining was used for evaluating CCT7 and the immunological T cell subset marker CD8α levels in cancer tissue samples of LUAD patients. After dehydration and embedding of LUAD cancer tissues to make sections, dewaxing was carried out. Antigen repair was performed using 3% citric acid repair solution and 3% H_2_O_2_ was used to block endogenous peroxidase. Next, BSA was added to prevent non‐specific binding, then diluted CCT7 and CD8α primary antibodies were added and incubated overnight under 4°C. After primary antibody staining, the corresponding fluorescent secondary antibody mixed working solution (Cat# ZF‐0512, ZF‐0512; ZSGB‐bio, China) was dropped in, and finally the DAPI staining solution (Cat# AR1177; BosterBio, China) was added to incubate away from light under ambient temperature to complete staining. Images were collected using a fluorescence inverted microscope. The primary antibodies used were respectively: CD8α antibody (Cat# 66868‐1‐Ig, 1:400, Proteintech); CCT7 antibody (Cat# ab166891, 1:100, Abcam).

### 2.12 Statistical Analysis

Bioinformatics analysis was implemented with R software (version 4.3.2) for statistical analyses and visualization. We adopted GraphPad Prism 9.0 (GraphPad Software, USA) to implement statistical analyses. Between‐group significance was analyzed by unpaired *t*‐test. *p* < 0.05 stood for statistical significance.

## 3 Results

### 3.1 High CCT7 Expression Within LUAD

**FIGURE 1:**
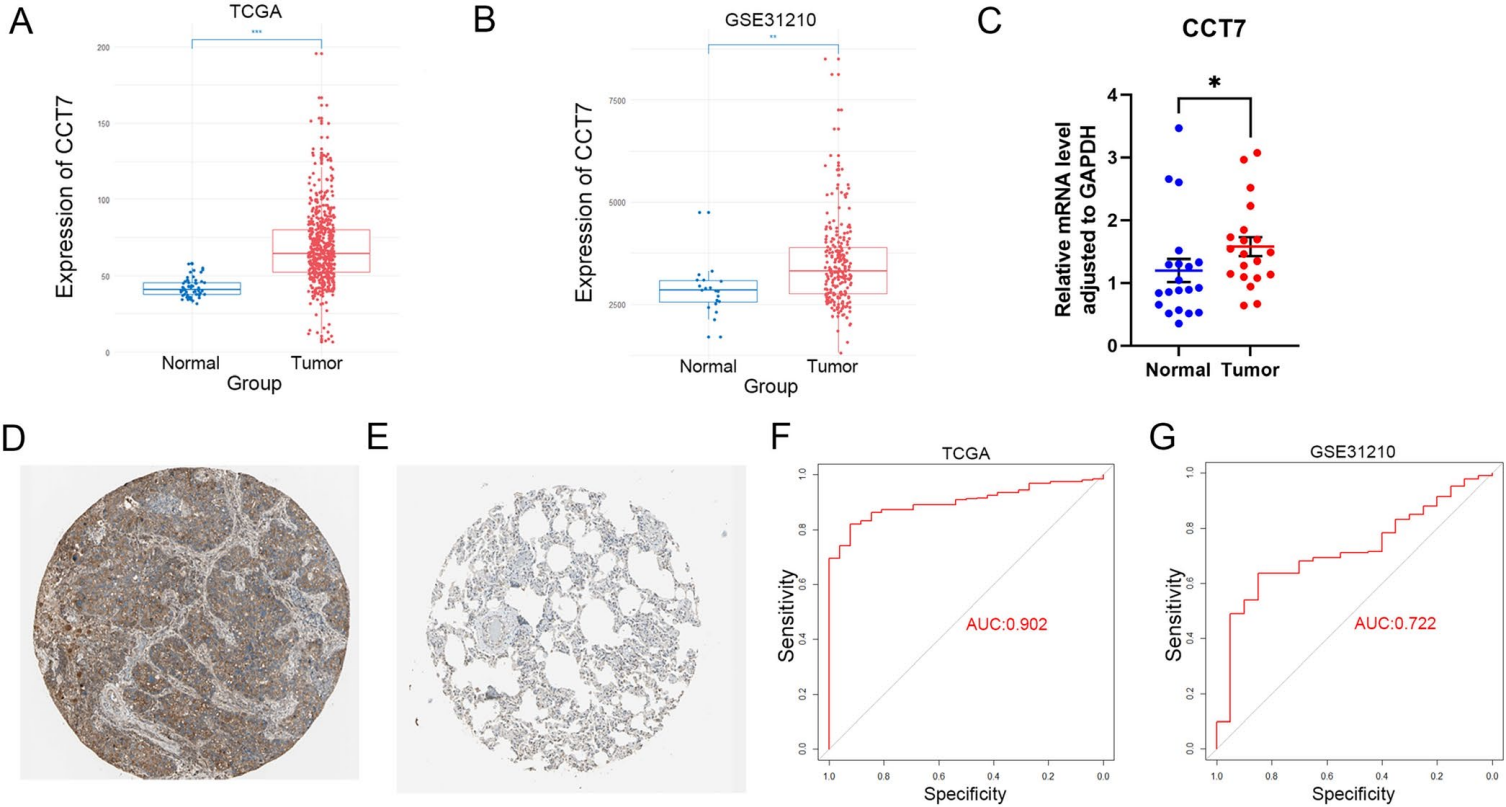
Expression of CCT7 in normal lung tissue and LUAD tissue. (A) Expression of CCT7 in LUAD tissues compared to normal lung tissues in the TCGA dataset. (B) Expression of CCT7 in LUAD and normal lung tissues in the GSE31210 dataset. (C) Expression of CCT7 in tumor tissues and adjacent normal tissues of LUAD patients. (D) Protein expression of CCT7 in LUAD tissues according to the HPA database. (E) Protein expression of CCT7 in normal lung tissues according to the HPA database. (F) Diagnostic ROC curve for CCT7 in the TCGA dataset. (G) Diagnostic ROC curve for CCT7 in the GSE31210 dataset.

To compare CCT7 expression within LUAD tissues and non‐carcinoma lung samples, CCT7 expression was analyzed in the TCGA‐LUAD cohort. As discovered, CCT7 expression markedly increased within LUAD relative to non‐carcinoma lung samples (*p* < 0.001, Figure 1A). The validation dataset (GSE31210) was analyzed, indicating higher CCT7 expression within LUAD than non‐carcinoma lung samples (*p* < 0.01, Figure 1B). Additionally, CCT7 expression was analyzed by RT‐qPCR in LUAD and matched non‐carcinoma lung samples in 20 LUAD cases. As a result, CCT7 was significantly overexpressed inside LUAD samples (*p* < 0.05, Figure 1C). HPA database‐derived immunohistochemical staining results demonstrated increased CCT7 protein levels within LUAD samples relative to non‐carcinoma lung samples (Figure 1D,E).

We employed ROC curve analysis to distinguish LUAD from non‐carcinoma lung samples. The AUCs for the TCGA (Figure 1F) and GSE31210 (Figure 1G) datasets were 0.902 and 0.722, which indicated that CCT7 expression can effectively distinguish LUAD tissues from normal lung tissues, making CCT7 the candidate marker to diagnose LUAD.

### 3.2 CCT7 Levels Are Related to Clinical Pathological Characteristics and LUAD Patient Prognosis

**FIGURE 2:**
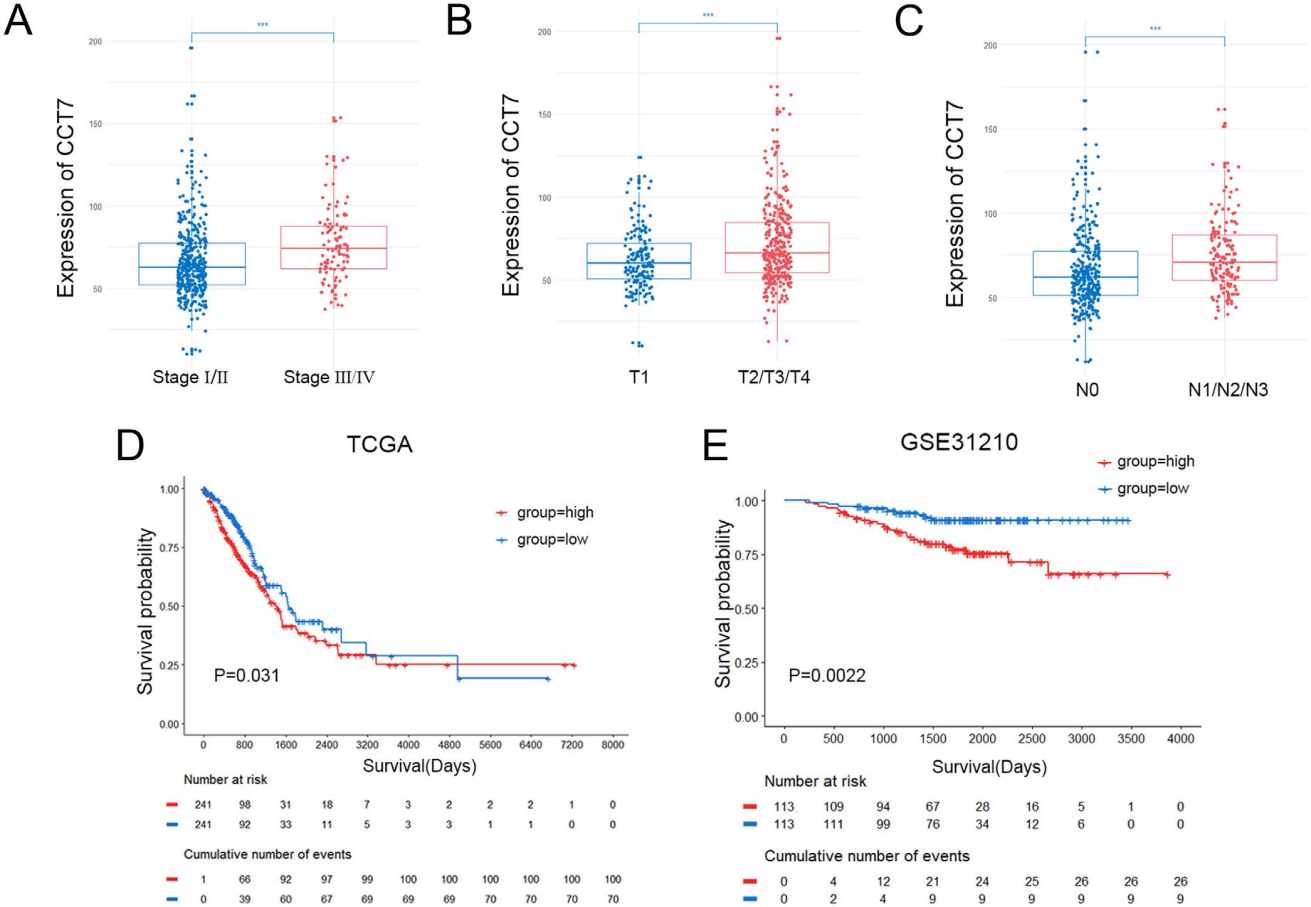
CCT7 expression in LUAD patients with different clinical pathological features. (A) CCT7 expression in LUAD patients with different pathological stages. (B) CCT7 expression in LUAD patients with different T stages. (C) CCT7 expression in LUAD patients with different N stages. (D) KM survival analysis of two groups with different CCT7 expression in the TCGA dataset. (E) KM survival analysis of two groups with different CCT7 expression in the GSE31210 dataset.

The TCGA‐LUAD cases with different clinical pathological features had different expressions of CCT7. The analysis indicated that LUAD patients with a higher pathological stage (Stage III/Stage IV) showed increased CCT7 expression compared with those with a lower pathological stage (Stage I/Stage II, *p* < 0.001, Figure 2A). CCT7 expression was higher in LUAD patients at stages T2/T3/T4 compared to those at stage T1 (*p* < 0.001, Figure 2B). For those developing lymph node metastasis (N1/N2/N3), CCT7 expression increased relative to those not experiencing this condition (N0) (*p* < 0.001, Figure 2C).

KM curves were adopted for evaluating the relation of CCT7 expression with LUAD patient survival. The end event for patients was death, and differences between the two survival curves were compared by the Log‐rank test. The analysis showed that in the TCGA database, the OS of LUAD patients in the high CCT7 expression group was significantly shorter than that of patients in the low CCT7 expression group (*p* = 0.031, Figure 2D). The GSE31210 dataset verified the above result (*p* = 0.0022, Figure 2E). According to these findings, CCT7 upregulation is related to shortened OS and unfavorable prognostic outcomes of LUAD patients.

### 3.3 Establishment of OS Prediction Nomogram

**FIGURE 3:**
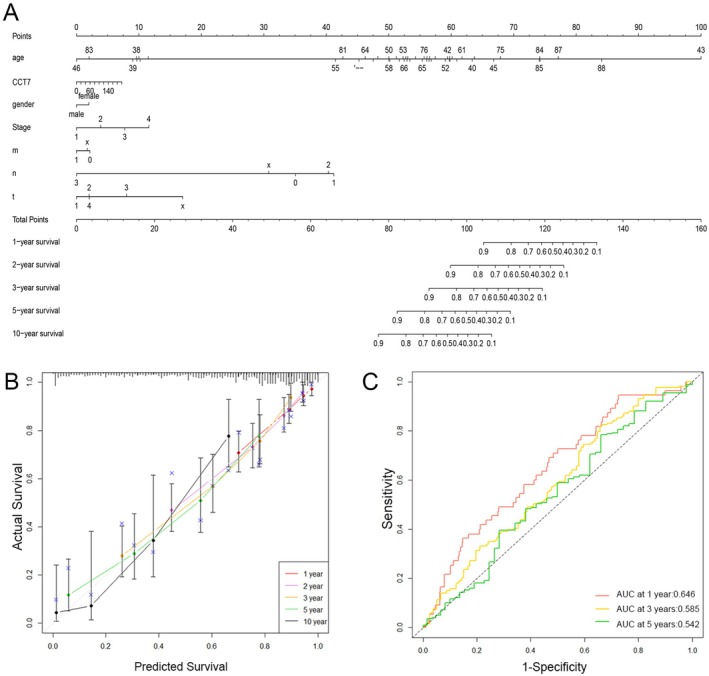
Nomograms for predicting OS in LUAD patients. (A) Nomogram for predicting the 1‐, 2‐, 3‐, 5‐, and 10‐year survival rates of LUAD patients. (B) Calibration curves for nomogram of the 1‐, 2‐, 3‐, 5‐, and 10‐year survival rate. (C) ROC curves and AUC for the 1‐, 3‐, and 5‐year survival rates of the nomogram.

The OS prediction nomogram was established for LUAD patients using clinical information including CCT7 expression, gender, age, and TNM staging (Figure 3A). For OS, its c‐index was 0.758, indicating good discriminatory ability of the nomogram. The actual observed vs. predicted ratios for 1‐, 2‐, 3‐, 5‐, and 10‐year OS were closely matched on calibration curves (Figure 3B). The AUCs for 1‐, 3‐, and 5‐year survival rates of LUAD cases were determined to be 0.646, 0.585, and 0.542 (Figure 3C).

### 3.4 Functional Enrichment Analysis

**FIGURE 4:**
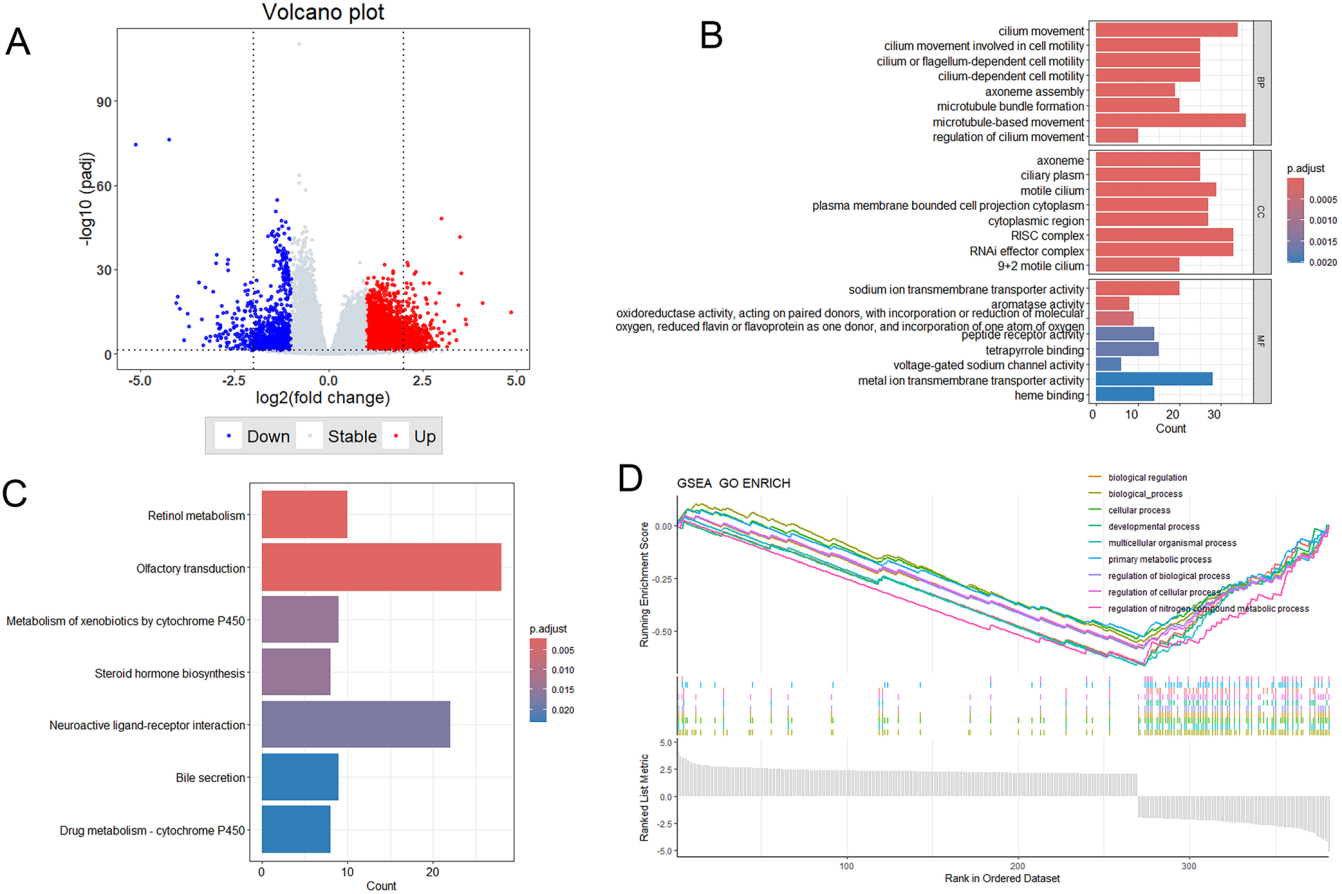
Functional analysis of CCT7. (A) Volcano plot showing DEGs between groups with different CCT7 expression in the TCGA‐LUAD cohort. (B) Results of GO enrichment analysis for DEGs in LUAD patients with different CCT7 expression. (C) Results of KEGG enrichment analysis for DEGs in LUAD patients with different CCT7 expression. (D) Results of GSEA enrichment analysis for DEGs in LUAD patients with different CCT7 expression. The length of the bars indicates the degree of gene enrichment, while the color of the bars represents significance.

In line with median CCT7 expression, we divided TCGA‐LUAD patients in high or low CCT7 expression group, and analyzed DEGs in these two groups. Using the |logFC| ≥ 1.0 and *p*_adj_ < 0.05 thresholds, we obtained 5194 DEGs, and constructed a volcano plot to visualize the results (Figure 4A). Thereafter, these DEGs were subjected to functional enrichment. As revealed by GO analysis depicted in Figure 4B, CCT7 was related to biological events like cytoskeleton organization and cell motility, constitutes cellular components including microtubule‐related processes, and was associated with molecular functions such as “aromatase activity”, “sodium ion transmembrane transporter activity”, “metal ion transmembrane transporter activity”. In KEGG enrichment, these DEGs were associated with pathways such as “retinol metabolism”, “olfactory transduction”, “metabolism of xenobiotics by cytochrome P450”(Figure 4C). Additionally, from GSEA results, DEGs were closely associated with pathways like “biological regulation”, “biological process”, “cellular process”, “developmental process”, and “multicellular organismal process”, indicating that CCT7‐related DEGs were primarily related to biological events including cellular metabolism and cellular composition (Figure 4D).

### 3.5 CCT7‐Associated Genes Within LUAD Cohort

**FIGURE 5:**
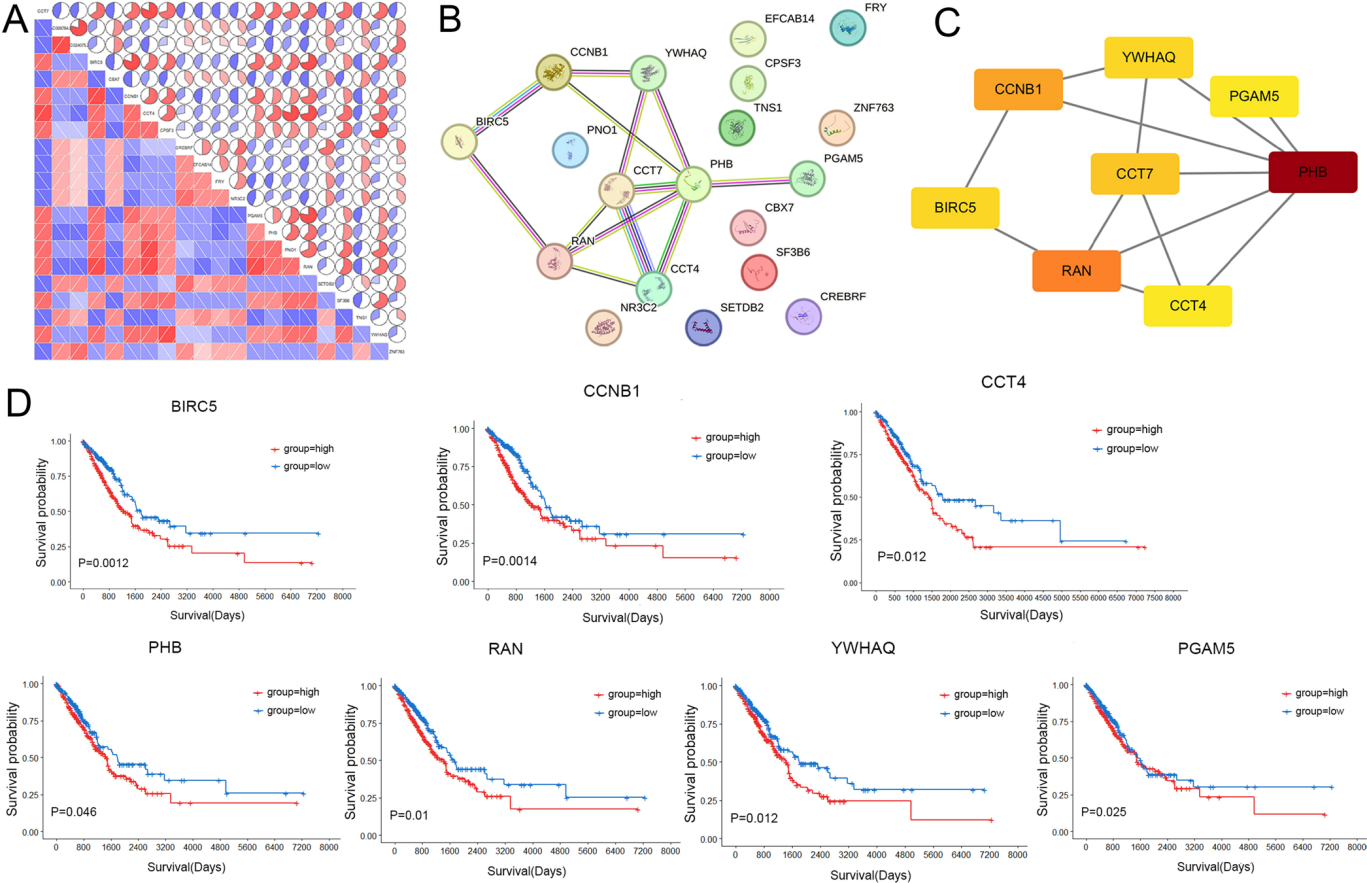
Correlation analysis of CCT7‐related genes in the TCGA‐LUAD cohort. (A) The top 20 genes most correlated with CCT7 and their correlations. (B) PPI interaction network of CCT7 and related genes. (C) Visualization of the PPI interaction network. (D) KM survival analysis of CCT7‐related genes.

Using Pearson's test, CCT7‐associated genes in TCGA‐LUAD cohort were subjected to correlation analysis, identifying the top 10 genes showing highest positive relation with CCT7 and 10 with highest negative relation to CCT7 (Figure 5A). STRING database was utilized for constructing a PPI network for identifying interactions between CCT7 and related genes, revealing that PHB, YWHAQ, RAN, and CCT4 have interactions with CCT7 (Figure 5B). The entire PPI network contains 19 nodes and 13 edges. The PPI network was monitored with CytoNCA plugin in Cytoscape software (version 3.10.1). Genes with a high frequency of interactions were considered hub genes within this PPI network, such as PHB, RAN, and CCNB1 (Figure 5C). From survival analysis, upregulation of other associated genes in the PPI network was related to poor survival of LUAD patients except for PGAM5 (*p* < 0.05, Figure 5D).

### 3.6 Relation of CCT7 Expression With Immune Infiltration in LUAD

**FIGURE 6:**
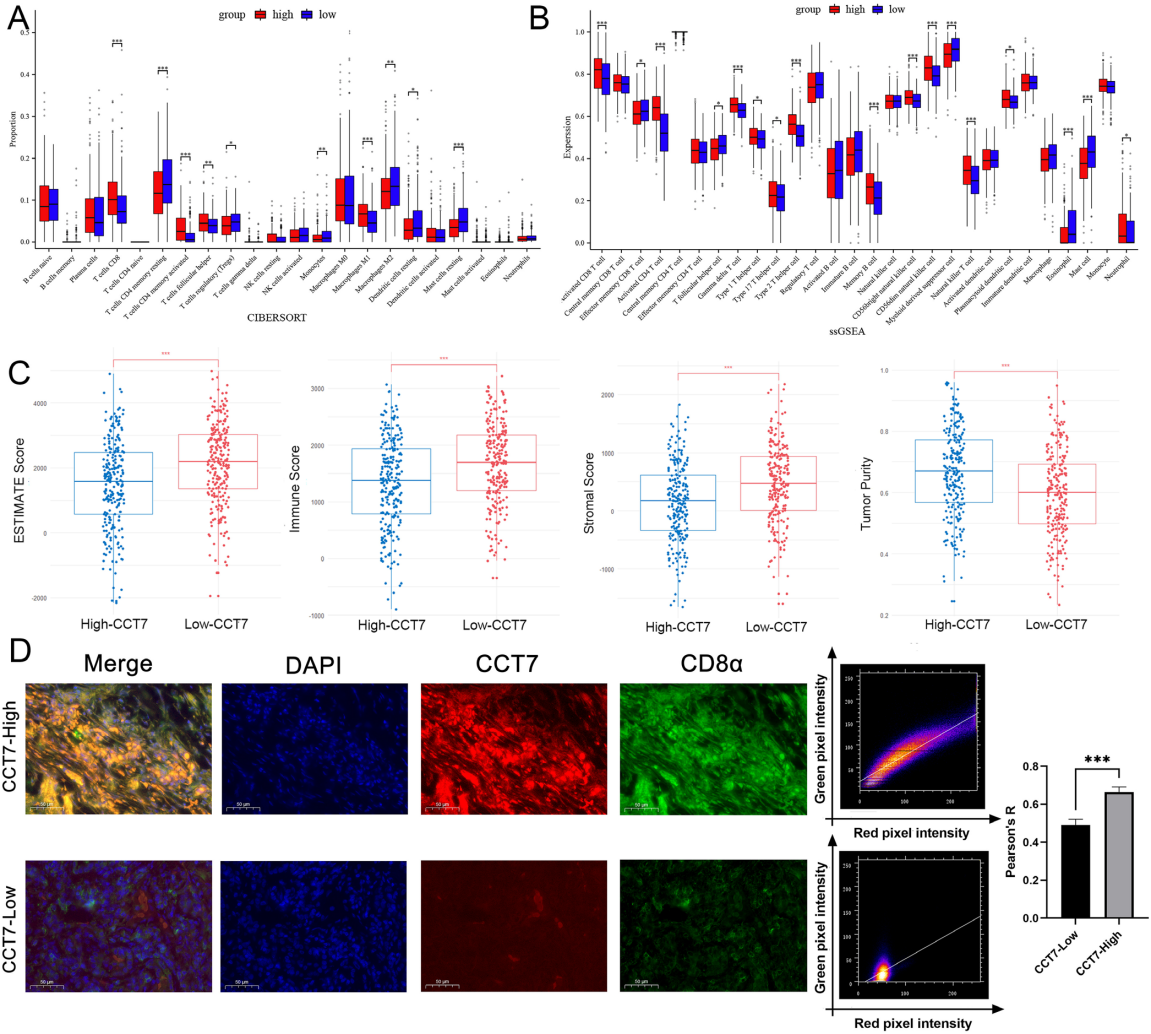
The relationship between CCT7 expression and immune infiltration in LUAD patients. (A) CIBERSORT analysis results of LUAD patients with different CCT7 expression. (B) ssGSEA analysis results of LUAD patients with different CCT7 expression. (C) Analysis of ESTIMATE score, immune score, stromal score, and tumor purity in LUAD patients with different CCT7 expression. (D) Results of co‐localization multiplex immunofluorescence staining of CCT7 and CD8α in cancer tissues of LUAD patients.

We conducted CIBERSORT and ssGSEA analyses and calculated ESTIMATE scores for different groups to compare their differences, so as to analyze the relation between CCT7 expression and immune infiltration among LUAD patients. CIBERSORT results indicated that CCT7 expression showed a positive relation with four tumor‐infiltrating immune cell subtypes, including activated memory CD4^+^ T cells, CD8^+^ T cells, T follicular helper cells, and M1 macrophages; besides, it showed a negative correlation with six tumor‐infiltrating immune cell subtypes: resting memory CD4^+^ T cells, T regulatory cells (Tregs), monocytes, M2 macrophages, resting dendritic cells, and resting mast cells (Figure 6A). ssGSEA results demonstrated that CCT7 expression was positively correlated with the expression of 12 tumor‐infiltrating immune cell subtypes: activated CD8^+^ T cell, activated CD4^+^ T cell, gamma delta T cell, T helper type 1 cell, T helper type 17 cell, T helper type 2 cell, memory B cell, CD56 (bright) natural killer cell, CD56 (dim) natural killer cell, natural killer T cell, plasmacytoid dendritic cell, and neutrophil; whereas inversely related to 5 tumor‐infiltrating immune cell subtypes: effector memory CD8^+^ T cell, T follicular helper cell, eosinophil, myeloid‐derived suppressor cell, and mast cell (Figure 6B). The high CCT7 expression group showed decreased immune/stromal/ESTIMATE scores, but greater tumor purity (*p* < 0.001, Figure 6C). To confirm the relation of CCT7 expression with immune cell infiltration, co‐expression of CCT7 with CD8+ T cell marker CD8α was assessed using mIF staining. As suggested by mIF analysis, CD8α expression within the cancer samples in LUAD patients having high CCT7 expression was relatively higher, and the expression positions of CCT7 and CD8α were more consistent and the expression correlation was higher, demonstrating that LUAD cases having increased CCT7 expression were associated with increased CD8+ T cell infiltration (Figure 6D).

### 3.7 Differences of TMB and Expression of Immune Checkpoint Between Two Groups

**FIGURE 7:**
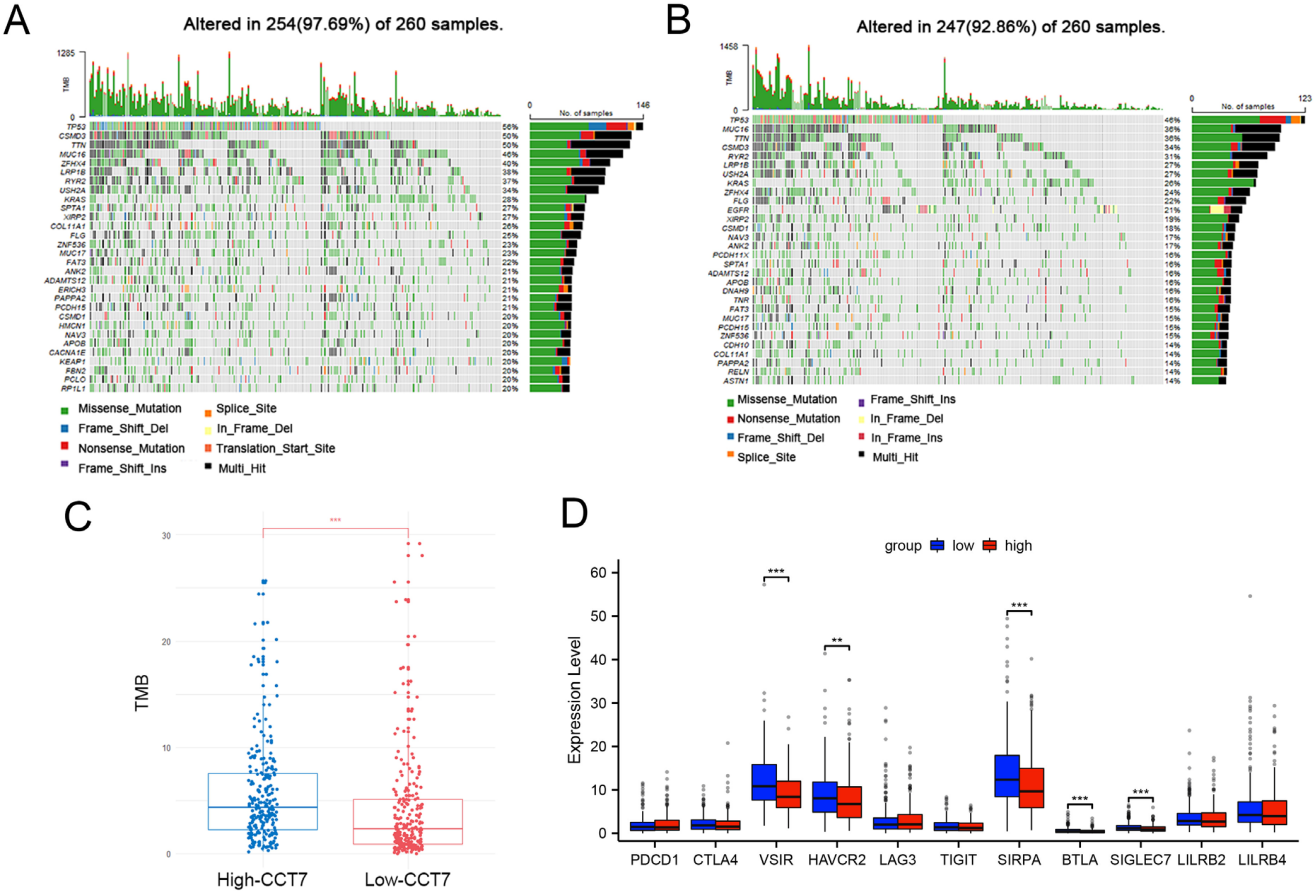
Analysis of TMB and expression of immune checkpoint between different CCT7 expression groups. (A) Gene mutation map of LUAD patients in the high CCT7 expression group. (B) Gene mutation map of LUAD patients in the low CCT7 expression group. (C) TMB differences in patients with LUAD expressed by different CCT7 expression groups. (D) Differences in the expression of 11 immune checkpoints between different CCT7 expression groups.

Immune checkpoint levels and genetic mutations influence the tumor immunotherapeutic effects. We evaluated genetic mutation profiles in TCGA‐LUAD patients who had different CCT7 expression levels. Patients showing high CCT7 expression carried more genetic mutations than those having low expression, especially more mutations in TP53. The waterfall plots in Figure 7A,B display those 30 most mutated genes of both groups, and patients having high CCT7 expression had greater TMB scores (Figure 7C). Besides, differences in 11 immune checkpoint levels were compared, finding that five immune checkpoints including VSIR, HAVCR2, SIRPA, BTLA, and SIGLEC7 had lower levels among those showing high CCT7 expression than those exhibiting low expression (Figure 7D).

## 4 Discussion

Our study confirms that CCT7 is overexpressed within LUAD samples, which has good diagnostic value for distinguishing between LUAD tissues and normal lung tissues. Furthermore, our findings indicate that patients of later pathological stage, higher T stage, and lymph node metastasis have higher CCT7 expression. From survival analysis, those having higher CCT7 expression have shorter OS, which suggests that CCT7 expression may predict unfavorable prognostic outcomes of LUAD patients.

As a subunit of CCT, CCT7, like other subunits of the CCT, can facilitate cell motility by improving the cytoskeleton proteins actin and tubulin binding sites [12]. CCT7's molecular effects on LUAD were analyzed by GO, KEGG, along with GSEA analysis. As confirmed by GO as well as KEGG analysis, CCT7 was associated with cell motility and microstructure, involved in the formation of tubulin, and in processes associated with microtubules and related to cell motility. The functional enrichment analysis results indicate that CCT7 impacts cell cycle and promotes LUAD tumor progression by affecting cell motility and structure. From KEGG analysis, CCT7 was related to cytochrome P450 and drug metabolism involving P450. Cytochrome P450 is related to the development of LUAD [13]. This further suggests that CCT7 is related to LUAD occurrence and development. GSEA enrichment analysis indicated that CCT7 was associated with a variety of cellular processes. The results of these functional analyses all hint at CCT7's indispensable effect on LUAD occurrence and development.

Through Pearson's test analysis and PPI network construction, we identified seven genes related to CCT7, with PHB, YWHAQ, RAN, and CCT4 showing the closest association with CCT7. According to a pan‐cancer analysis, PHB shows overexpression within both LUAD and LUSC tissues and is associated with unfavorable survival of LUAD cases [14]. The study by Raungrut et al. [15] noted that overexpression of YWHAQ could promote the invasion of NSCLC and lead to dismal survival of NSCLC patients, serving as the potential prognostic biomarker for advanced lung cancer. Additionally, there are researchers utilizing immunoprecipitation who identified YWHAQ‐associated proteins in NSCLC cells, revealing that YWHAQ and these proteins are involved in NSCLC migration and invasion, and are potential therapeutic targets for NSCLC [16]. RAN has been discovered to with upregulation inside NSCLC cells, which activates the PI3K‐AKT pathway to enhance NSCLC cell migration, revealing RAN as the candidate anti‐NSCLC therapeutic target [17]. Li et al. [18] found that CCT4 can interact with CDC20 and participate in liver cancer pathogenesis. Based on Wang et al. [19], CCT4 regulates the ErbB pathway and affects the development and progression of Wilms' tumor, indicating that CCT4 is related to tumorigenesis.

Tumor occurrence is linked to their surrounding environment and tumor microenvironment (TME). Typically, the TME includes tumor cells, immune cells (like B cells, T cells, dendritic cells), adipocytes, cytokines, and exosomes, displaying complex components. The increase in immune cells inside the TME disrupts lung immune homeostasis, facilitating the progression and metastasis of NSCLC [20]. The immune cells in the TME might influence cancer genesis and progression; also, immune cell infiltration levels are associated with disease metastasis and treatment resistance. Specifically, the abundance of T lymphocytes is linked to tumor patient survival [21, 22]. Diverse immune cell infiltration patterns influence clinical efficacy for patients to varying degrees. Tumors infiltrated by T cells may respond to treatments targeting immunosuppressive mechanisms. Tumors infiltrated by non‐T cells probably obtain other interventions for promoting innate immune activation and inflammation within the TME. Macrophage, T helper cell, and dendritic cell infiltration at tumor invasion front and margin is associated with radio‐ and chemoresistance and shorter survival times [23, 24]. Building on our preliminary functional analyses, the interaction of CCT7 expression with tumor immune infiltration within LUAD was also analyzed. From our ssGSEA and CIBERSORT results, differential CCT7 expression was primarily observed in T cells, hypothesizing that the two groups of patients had different immune backgrounds. The existing studies usually indicate that the high M2 macrophage and Treg infiltration is correlated with unfavorable survival among cancer cases [25]. The existence of Tregs is significant for immunity and tumor occurrence. Tregs can achieve immune tolerance of tumors by regulating the immune factor levels (IL‐10, TGF‐β) to promote tumor development [26]. M2 macrophages are generally considered to be carcinogenic and help the tumor to achieve immune escape by releasing cytokines such as inflammatory factors and chemokines and promoting the formation of microvascular in TME [27]. Interestingly, we observed that the infiltration levels of Tregs and M2 macrophages in those having low CCT7 expression markedly decreased relative to those showing high expression. Furthermore, in our ESTIMATE analysis, relative to patients exhibiting low CCT7 expression, LUAD patients with increased CCT7 expression showed lower ESTIMATE scores, lower immune and stromal cell infiltration levels, whereas greater tumor purity; these results suggest the complicated association of CCT7 expression with the TME of LUAD patients. Similar research results have been reported before [28].

TMB is associated with the quantity of tumor mutations, and cancer patients with higher TMB tend to have more effective responses to immunotherapy [29]. Our analysis revealed that LUAD patients having increased CCT7 expression showed an elevated TMB relative to those having decreased CCT7 expression. Moreover, high CCT7 expression patients also exhibited a higher gene mutation rate, particularly with mutations in TP53, CSMD3, and TTN occurring in over 50% of the high CCT7 expression group. TP53 is related to biological events including DNA repair, autophagy, apoptosis, cell cycle arrest, cellular senescence, and metabolism, and its inactivation or dysfunction is a hallmark of cancer. Mutations in TP53 are often associated with cancer onset, lower survival rates in patients, and treatment resistance [30]. Further studies indicate that patients with high TP53 mutation burdens have higher TMB [31], as one of the genes with the highest mutations in NSCLC, TP53 mutations significantly shorten OS in LUAD cases [32]. Noteworthy, a single‐cell sequencing study has indicated that in a high‐risk model of a Tregs marker gene, patients with LUAD also had low Tregs infiltration, high TMB score, and low immune score, and these patients also exhibited a poorer prognosis [33], which is similar to our research results. These studies have revealed the complex immune environment of LUAD patients. The effectiveness of the immune system in defending against tumors not only depends on the number of cells that penetrate the TME but also on their functional status [34]. Our research results suggest that CCT7 may, to some extent, cause immune cell dysfunction among LUAD patients. Prognosis of LUAD patients may also be more associated with immune cells' functional status, indicating that more comprehensive and integrated analysis is needed for exploring how immune cell infiltration level predicts LUAD prognosis. According to our results, for LUAD patients showing increased CCT7 expression, the levels of expression for immune checkpoints (VSIR, HAVCR2, SIRPA, BTLA, SIGLEC7) were generally lower. These immune checkpoints are critical regulators of immune homeostasis and anti‐tumor immunity [11], suggesting that those having increased CCT7 expression experience deeper immune homeostasis disruption, which could translate to an increased likelihood of tumor metastasis and migration. This also explains why our high CCT7 expression group patients present with higher pathological stages and more lymph node metastasis. Additionally, as CCT7 is a protein folding and assembly factor, it may regulate the stability or activity of certain immune regulatory factors within tumor cells, thereby affecting some immune checkpoint levels. However, specific details of this mechanism still require further investigation to clarify. In summary, our study suggests that CCT7 is involved in the formation of a complex TME in LUAD patients, which sheds novel light on individualized and precise anti‐LUAD treatments. Nevertheless, the specific mechanisms of the relationship between CCT7 and immunoregulation still need to be further investigated to provide a more reliable theoretical basis for clinical treatment.

## 5 Conclusions

In conclusion, CCT7 expression increases within LUAD, which leads to unfavorable outcomes of LUAD cases, suggesting CCT7's key impact on LUAD occurrence and development. CCT7 may be the potential anti‐LUAD therapeutic target, presenting further opportunities for prognosis improvement in LUAD patients.

## Author Contributions

**Yue Li:** methodology, writing – original draft, writing – review and editing, conceptualization, investigation. **Longqian Wei:** investigation, writing – review and editing, writing – original draft, formal analysis. **Guiyu Feng:** writing – review and editing, writing – original draft, data curation, validation. **Guosheng Li:** visualization, methodology, software. **Jielin Ma:** investigation, visualization. **Xiang Gao:** formal analysis, methodology. **Jun Liu:** project administration, resources, supervision. **Nuo Yang:** resources, project administration, supervision. **Huafu Zhou:** project administration, resources, supervision.

## Funding

This work was supported by the National Key Clinical Specialty Construction Project; Guangxi Medical and Health Key Discipline Construction Project; Guangxi Key Clinical Specialty Construction Project.

## Ethics Statement

This study was performed in line with the principles of the Declaration of Helsinki. Approval was granted by the Ethics Committee of the First Affiliated Hospital of Guangxi Medical University (no. 2024‐E338‐01).

## Consent

The informed consent was obtained from all subjects and their legal guardians.

## Conflicts of Interest

The authors declare no conflicts of interest.

## Data Availability

The RNA transcriptome data and clinical data used in this study were obtained from The Cancer Genome Atlas (TCGA) database (https://portal.gdc.cancer.gov/) LUAD cohort (TCGA‐LUAD), Gene Expression Omnibus (GEO) database (https://www.ncbi.nlm.nih.gov/geo/), and HPA database (https://www.proteinatlas.org/).
